# A label-free differential quantitative mass spectrometry method for the characterization and identification of protein changes during citrus fruit development

**DOI:** 10.1186/1477-5956-8-68

**Published:** 2010-12-16

**Authors:** Ehud Katz, Mario Fon, Richard A Eigenheer, Brett S Phinney, Joseph N Fass, Dawei Lin, Avi Sadka, Eduardo Blumwald

**Affiliations:** 1 Department of Plant Sciences, University of California, Davis, CA, 95616, USA; 2 Genome Center, Proteomics Core Facility, University of California, Davis, CA, 95616, USA; 3 Genome Center, Bioinformatics Core Facility, University of California, Davis, CA 95616, USA; 4 Department of Fruit Tree Species, ARO, The Volcani Center, 50250 Bet Dagan, Israel

## Abstract

**Background:**

Citrus is one of the most important and widely grown commodity fruit crops. In this study a label-free LC-MS/MS based shot-gun proteomics approach was taken to explore three main stages of citrus fruit development. These approaches were used to identify and evaluate changes occurring in juice sac cells in various metabolic pathways affecting citrus fruit development and quality.

**Results:**

Protein changes in citrus juice sac cells were identified and quantified using label-free shotgun methodologies. Two alternative methods, differential mass-spectrometry (dMS) and spectral counting (SC) were used to analyze protein changes occurring during earlier and late stages of fruit development. Both methods were compared in order to develop a proteomics workflow that could be used in a non-model plant lacking a sequenced genome. In order to resolve the bioinformatics limitations of EST databases from species that lack a full sequenced genome, we established iCitrus. iCitrus is a comprehensive sequence database created by merging three major sources of sequences (HarvEST:citrus, NCBI/citrus/unigenes, NCBI/citrus/proteins) and improving the annotation of existing unigenes. iCitrus provided a useful bioinformatics tool for the high-throughput identification of citrus proteins. We have identified approximately 1500 citrus proteins expressed in fruit juice sac cells and quantified the changes of their expression during fruit development. Our results showed that both dMS and SC provided significant information on protein changes, with dMS providing a higher accuracy.

**Conclusion:**

Our data supports the notion of the complementary use of dMS and SC for label-free comparative proteomics, broadening the identification spectrum and strengthening the identification of trends in protein expression changes during the particular processes being compared.

## Background

Fruit ripening and development has being studied using transcriptomic, proteomics, and metabolomics approaches [1-8]. Quantitative proteomics provides an alternative approach for studies of fruit development. In the last few years, quantitative proteomics has been widely applied for the quantification of complex biological samples [9-11]. The most commonly used approach for comparative proteomic analysis of plant tissues is the application of 2DE-gels. This method is limited in sensitivity, has a low dynamic range, it is inefficient when analyzing insoluble proteins or proteins with very high or low molecular mass and are limited in their reproducibility [12],  although reproducibility has been improved with the use of differential imaging gel electrophoresis (DIGE) [13,14]. Alternative techniques to 2DE-gels are non-gel LC-MS/MS-based shotgun proteomics [15-18], where quantification is performed using the mass-spectrometer data. Some success for the quantification of proteins has been achieved by using stable isotope labeling, ^15^N, ^13^C, ^2^H and SILAC [19], ICAT [20,21], iTRAQ [22] and ^18^O stable isotope incorporation [23]. One of the main limitations of these methods is that full labeling of the proteins is rarely achieved and that different peptides incorporate the label at different rates which complicates data analysis. Recently, a label-free method for comparative proteomic analysis has emerged [9-11,24].

Label-free proteomics allows for the quantification of peptides using spectral characteristics such as retention time, m/z ratio and peak intensity by comparing the direct mass spectrometric signal intensity for any given peptide (differential Mass Spectrometry, dMS) or by counting the number of acquired tandem mass spectra matching to a specific peptide as an indicator for their abundance in a given sample (spectral counting, SC) [25,26]. dMS is based on comparisons of chromatographic peaks of peptide precursor ion measurements belonging to a specific protein extracted from an LC-MS/MS run [27-32]. This approach is based on the observation that dMS in most cases is proportional to the concentration of the peptide in the sample investigated [10,27-29]. Peak intensity for every individual spectrum is determined and the comparison of spectra between multiple LC-MS runs provides quantitative measurement of thousands of peptides. From this massive data a selected list of differential peptides can be produced for subsequent fragmentation by LC-MS/MS for sequence determination and protein identification.  In order to match the massive spectra data according to retention time and precursor m/z characteristics various software have been developed. Once matched, expression ratio in peak intensity is calculated according to peak areas corresponding to the matched peptides. SC counting is based on counting and comparing the number of spectra identifying specific peptides of a given protein to assess relative protein abundance, also found to be in good correlation with protein abundance [15,30].

Proteomics has been used successfully to characterize and identify changes in plant protein compositions during different developmental stages [3,5,33,34], and proteomic comparative analysis of citrus fruits, mainly using 2DE-gels, have been published recently [35-38].

Label-free comparative proteomics is a relatively new approach that has been used successfully in different systems (humans, yeast, fly, etc.) [39-42], but its application in plants is scanty [26,43]. Using LC-MS/MS we recently analyzed soluble and enriced membrane fractions of mature citrus fruit to identity the proteome of fruit juice cells and classified these proteins according to their putative function according to known biosynthetic pathways [18]. Here, we describe a method for the use of label-free LC-MS/MS-based shotgun differential proteomics for the study of fruit development in Citrus, a non-model plant lacking a fully sequenced genome. The method combines the use of dMS and SC and the creation of iCitrus, a citrus fruit-specific database and interface, for the identification of the protein changes occurring during the development of citrus fruits.

## Results

### Citrus proteins annotations using iCitrus

**Figure 1:**
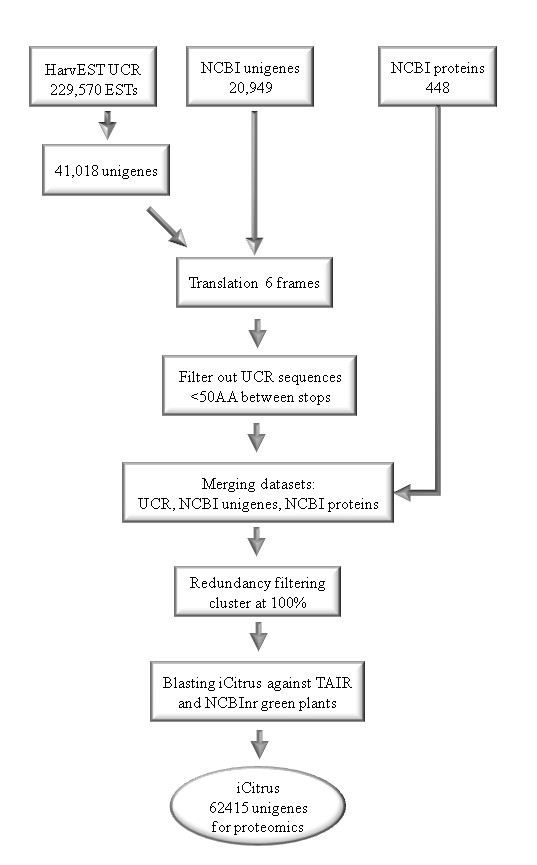
**iCitrus database**. Three major sources were used in creating iCitrus dataset: UC Riverside HarvEST:citrus (C46 assembly), NCBI/citrus/unigenes and NCBI/citrus/proteins (see text). The first two datasets were translated into all 6 reading frames, split at stop codons, and sequences shorter than 50 amino acids were removed. These were combined with the NCBI protein sequences, and all three protein sequence sets were then clustered at 100% identity using CD-HIT http://bioinformatics.ljcrf.edu/cd-hi/, meaning that sequences that aligned with 100% identity to a longer sequence in the combined set were removed. All remaining sequences were then blasted to TAIR proteins, and separately to the subset of NCBI's nr database belonging to taxa within *Viridiplantae*, to collect GO-term and descriptive annotation for the clustered sequences.

Although the citrus genome has not been fully sequenced yet, a comprehensive citrus EST database has been developed in the past few years [44].   Several groups have contributed to EST sequencing efforts using different species, including *C. sinensis *(sweet orange), *C. clementina *(Clementine mandarin), *C. paradisi *(grapefruit), *Poncirus trifoliata*, and other hybrids (*C. sinensis × Poncirus trifoliata*, Carrizo citrange). A wide range of libraries derived from multiple reproductive and vegetative tissues at different developmental stages were used in addition to different treatments or stresses to create a relatively large database. To date, there are 582,334 citrus sequences in the National Center for Biotechnology Information (NCBI) EST database. With the advantage of comprehensive sequence dataset in hand, there were many challenges to be addressed before using the databases for proteomic research. Some of these challenges arose from the nature of EST databases, over-representation of highly-expressed genes (and the underrepresentation of weakly-expressed genes), redundancy, incomplete sequences, poor annotation etc. The challenge of using the EST database for proteomics came from the fact that a highly redundant database with many similar sequences would artificially decrease the significance of potential "hits". On the other hand, a strong reduction in sequence-based redundancy, relying on sequence similarity rather than identity, would significantly reduce the number of possible hits. To solve some of these problems, iCitrus http://citrus.bioinformatics.ucdavis.edu/ was created (Figure 1). The iCitrus collected dataset was produced by excluding sequences shorter than 50 amino acids between stop codons and removing redundant sequences with 100% identity to another longer sequence in the dataset. Similar sequences, sharing less than 100% similarity were kept for spectra search. Keeping sequences sharing high similarity (97-99% identify) was a necessity because the citrus ESTs database consists of sequences originated from a wide range of citrus cultivars and species. Minor differences in nucleotide sequences between similar ESTs could lead to differences in amino acid sequences and therefore to differences in virtual spectra derived from the database during mass-spectra search. Keeping these sequences served to broaden our chances of identifying proteins in the databases while discarding them could lead to miss-identification or no identification of proteins. A disadvantage of this approach was the redundancy of accessions that were dealt with by manually aligning the sequences of the proteins of interest. In a few cases where the accessions shared a high similarity, the redundancy resulted in the identification of two or more ESTs with only one peptide. If these ESTs belong to the same unigene, then two or more peptides could identify the same specific protein.

To date, there are 62,415 sequences in the iCitrus collected database; 41,018 from the HarvEST:Citrus assembly http://harvest.ucr.edu/, 20,949 from NCBI's unigenes (C. *sinensis *and C. *clementina*), and 448 from NCBI's proteins (C. *sinensis *and C. *clementina*) (Figure 1). iCitrus dataset in a FASTA file format and a description of the iCitrus interface structure can be found as Additional File 1 and a conversion table  of HarvEST:Citrus, NCBI/Citrus/ESTs and NCBI/Citrus/Proteins accessions into iCitrus accessions can be found in Additional File 2: Table S1.

### Label-free LC-MS/MS based shotgun proteomics, differential Mass-Spec and Spectral Counting

**Figure 2:**
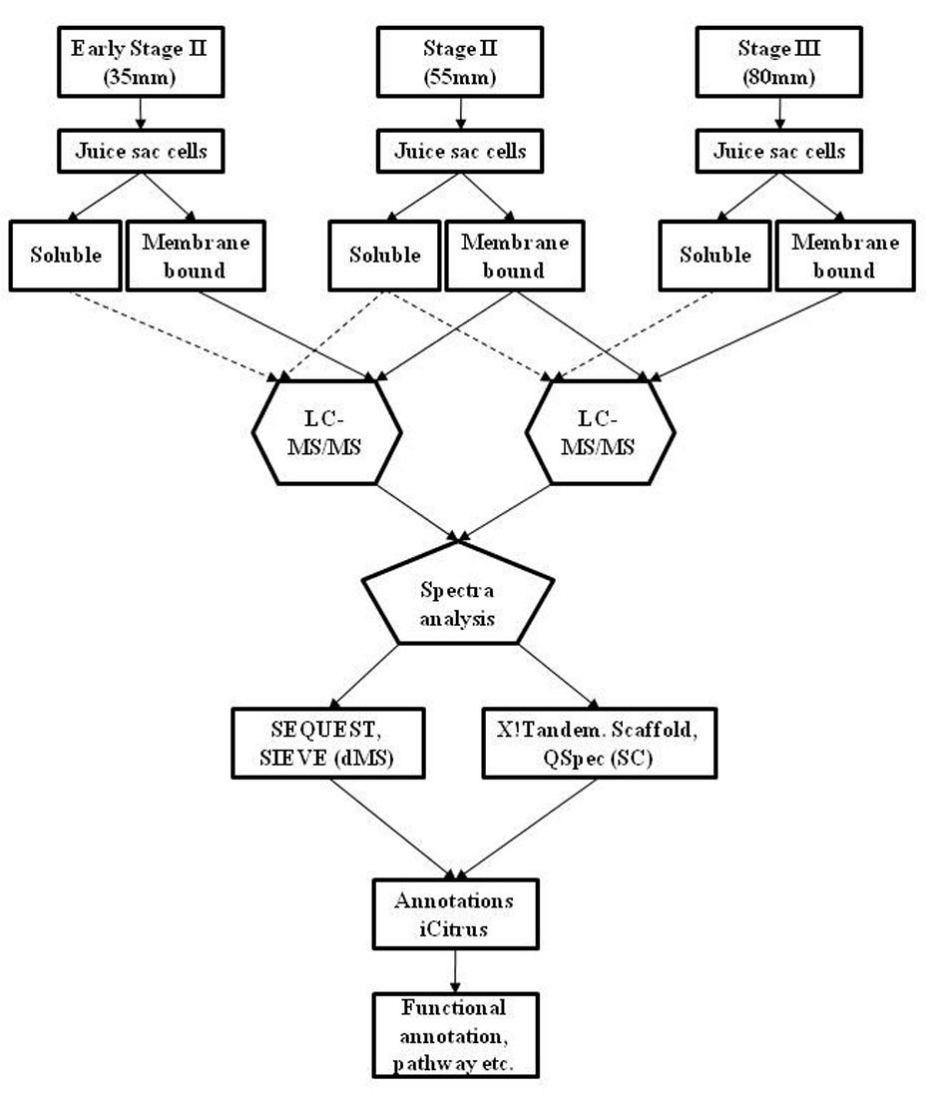
**Experimental design**. Soluble and membrane-bound proteins were extracted from juice sac cells from at least 20 fruits at three stages of Citrus fruit development (early Stage II, Stage II and Stage III) and pooled at each stage. Five technical repeats of each pooled sample (older *vs *younger fruit) were each analyzed by SIEVE using  blanks (washes) between each sample run.  Comparisons were conducted in pairs in the following: Stage II vs. early Stage II and stage III vs. Stage II. Methods as described in Experimental Procedures.

To achieve a better identification of differentially expressed proteins during fruit development and to decrease sample complexity, the juice sac cells were fractionated into soluble and membrane-bound proteins (Figure 2). Two alternative strategies for label-free mass spectrometric analysis; peptide ion intensities measurements and spectral counting were used.  The peptide ion intensities measurements, also referred as differential Mass Spec (dMS), integrate the peak area which is proportional to the concentration of the peptide in the sample (Additional File 3: Figure S1). Determining the area for each mass extracted peptide ion chromatogram retention time pair and comparing the areas between multiple LC-MS runs of different samples can provide a comprehensive quantification of thousands of peptides within samples. The alternative strategy, Spectral Counting (SC), calculates the number of MS/MS scans that are attributed to the same peptide ion. The frequency of these MS/MS scans correlates with the abundance of a given peptide in the sample. In this study we have used dMS strategy to analyze and identify differential proteins changes during fruit development in citrus juice sac cells (Figure 2) and SC as an alternative strategy to validate our finding. Identification of proteins was done by MS spectra search against the iCitrus database and annotations by using the iCitrus interface.

Label-free relative quantitative analysis detects, selects and compares spectra that are significantly different between samples (either by dMS or SC). However, many of the spectra that were selected as being different in their intensity or abundance were found to be not statistically different between the developmental stages compared and will be discussed later.

**Figure 3:**
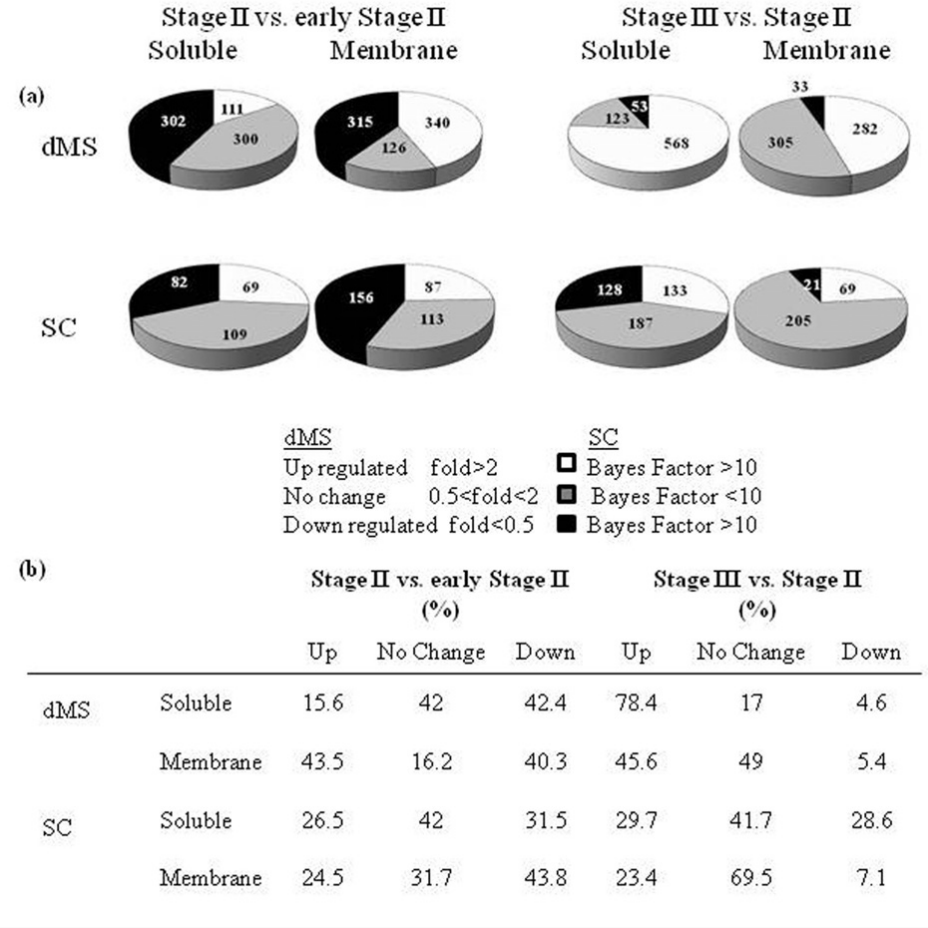
**Numbers of protein identified by dMS and SC**.

Using dMS, 1494 and 1364 proteins were identified by at least two peptides in the comparisons between Stage II (55 mm fruit diameter) versus early Stage II (35 mm fruit diameter) and Stage III (80 mm fruit diameter) versus Stage II, respectively (Figure 3). A high number of identified proteins were down- and up-regulated during the earlier and later stages of development, respectively (Figure 3a).

**Figure 4:**
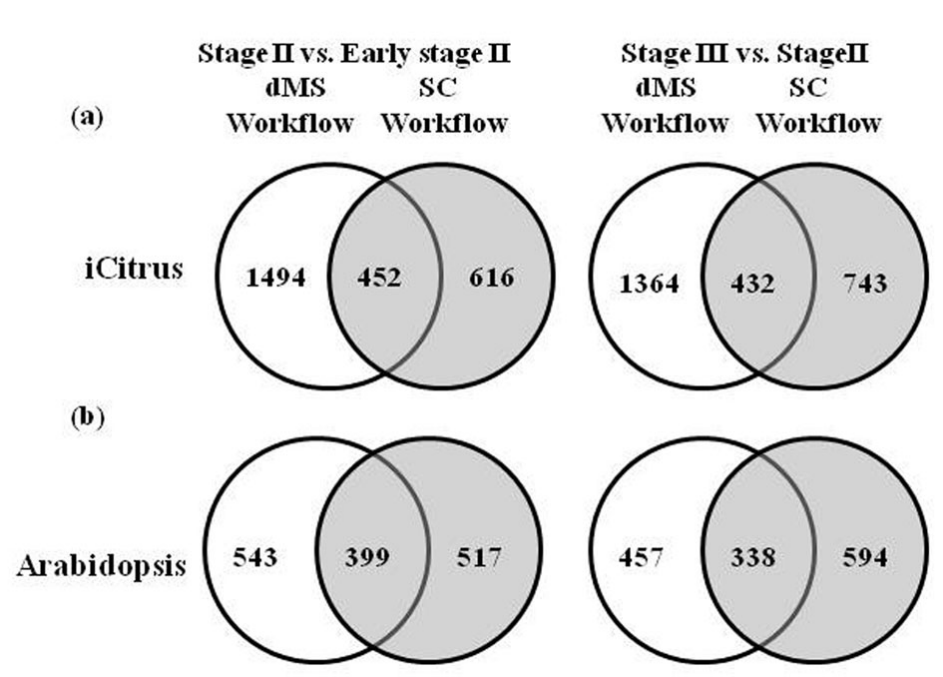
**Venn diagrams representing the number of proteins identified by both dMS and SC workflows and the number of proteins identified by only one of the workflows**. (a) Analysis was conducted by using iCitrus accessions of the identified proteins (b) Analysis was conducted by using the corresponding Arabidopsis homologs of the same iCitrus accessions presented in (a).

Accessions identified by SC and dMS were compared using both iCitrus and Arabidopsis homologs (Figure 4). These comparisons were made to minimize possible redundancies of identified citrus ESTs and to conserved citrus protein accessions that might originate from different unigenes but belonging to the same gene family. Once again, aconitase can provide a good example for database redundancy as the accessions 45840 and 47264, sharing 99% amino acid similarity, are essentially the same unigene originating from two different citrus species (Table 1). These accessions shared little similarity with 39802 and sequence alignment showed that their sequences did not overlap but shared high homology with the other members, i.e. 55395 and 43680. Notably, some proteins did not share homology to any Arabidopsis proteins, providing support to the use of citrus accessions for comparisons. In some cases, these accessions could be assembled to one contig while in other cases these ESTs could not be assembled. Two possibilities arose, either these EST sequences originated from the same gene but did not overlap, therefore could not be assembled, or these ESTs were originated from different genes belonging to the same family.

**Table 1:** Identification and quantification of aconitase by dMS and SC.

dMS			Stage II vs. early Stage II				Stage III vs. Stage II									
HarvEST Citrus ID	iCitrus ID	Blast Hit to TAIR ID	Ratio	Pvalue	No. peptides	Max. Xcorr	Ratio	Pvalue	No. peptides	Max. Xcorr						
UC46_7402	39802	AT2G05710	24.9	9.9E-20	3	5.39	33.6	3.621E-07	4	4.56						
UC46_5405	43680	AT2G05710	25.3	9.9E-20	2	5.39	13.6	9.9E-20	2	4.56						
UC46_5554	47264	AT2G05710	62.0	9.9E-20	2	5.40	4.9	0.0007414	3	6.52						
UC46_5555	45840	AT2G05710	49.7	9.9E-20	4	5.40	4.8	0.0014824	4	6.52						
UC46_9228	55395	AT2G05710	25.3	9.9E-20	2	5.39	9.4	4.115E-06	3	5.07						

**Spectral Counting**			**Stage II vs. early Stage II**							**Stage III vs. Stage II**						
HarvEST Citrus ID	iCitrus ID	Blast Hit to TAIR ID	Bayes Factor	Fold Change	Direction	No. peptides	-Log(e)	FDR Down	FDR up	Bayes Factor	Fold Change	Direction	No. peptides	-Log(e)	FDR Down	FDR up

UC46_7402	39802	AT2G05710	0.66	1.88	0	4	9.3	1.26	0.91	30.46	5.52	1	3	4.6	0.80	0.00
UC46_5405	43680	AT2G05710	-	-	-	-	-	-	-	143.42	12.24	1	2	3.7	1.03	0.88
UC46_5555	45840	AT2G05710	0.4	1.08	0	3	14.9	1.20	0.91	0.58	1.29	0	4	13.0	0.89	0.75
UC46_9228	55395	AT2G05710	0.42	1.08	0	2	11.7	1.26	0.91	0.68	1.11	0	3	11.0	0.73	0.86

	HarvEST		UC46_7402	UC46_5405	UC46_5555	UC46_5554	UC46_9228									
Origin	Citrus ID	iCitrus ID	39802	43680	45840	47264	55395									

C. Sinensis	UC46_7402	39802		87	17	18	86									
C. Sinensis	UC46_5405	43680			87	86	86									
C. Sinensis	UC46_5555	45840				99	90									
P. Trifoliata	UC46_5554	47264					89									
C. Sinensis	UC46_9228	55395														

All iCitrus accessions for aconitase that were identified by both methods were homolog to the Arabidopsis gene At2g05710. Identification of aconitase by dMS  and SC. The column "direction" under SC represents up-regulated = 1, no change = 0, down-regulated = -1.  Aconitase iCitrus accessions amino acids sequences similarities.

**Figure 5:**
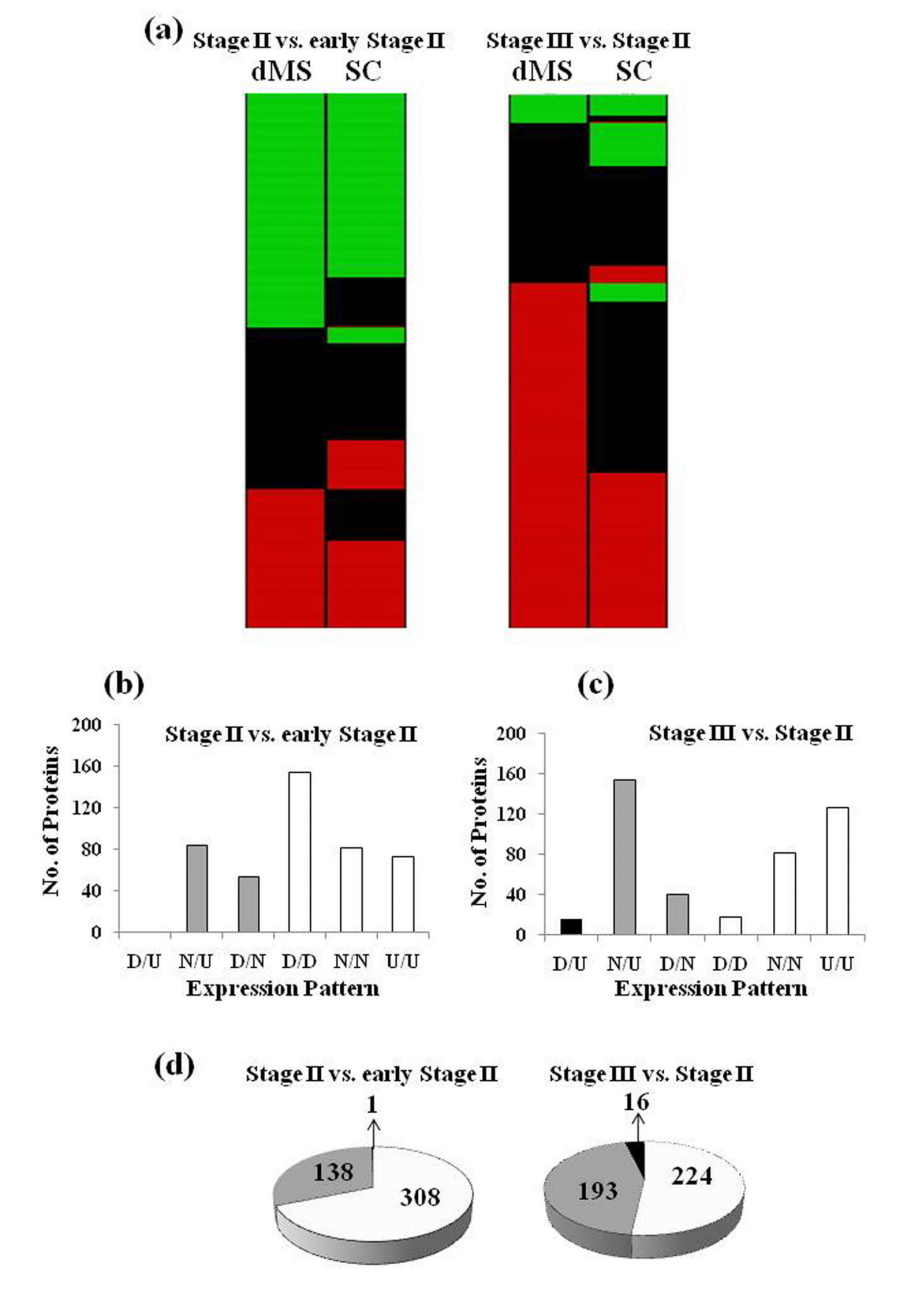
**Comparisons of expression trends of proteins identified by dMS and SC**. (a). Up-regulated (1-red), unchanged  (0-black),  down-regulated (-1-green).  (b-c) Proteins showing the same trend in dMS and SC (strong-match), contradicting trends (no-match) and "weak-match" for proteins identified by one of the methods as not changing. D-down-regulated, N-no change, U-up-regulated. Strong expression "match", (U/U, D/D, N/N according to dMS/SC) (white columns); "weak-match", (N/U, U/N, D/N, N/D by dMS/SC) (grey columns); "no-match", (D/U, U/D) (black columns); (b) Stage II vs. early Stage II (c) Stage III vs. Stage II. (d) Proteins identified by dMS and SC that have the same expression trend ("match", white), contradicting expression trend ("no-match", black) and  proteins up-regulated or down-regulated in one method but unchanged in the other ("weak-match", grey).

Most of the proteins identified by both dMS and SC also showed similar expression patterns (Figure 5). Out of 452 proteins identified by both methods in the comparison between fruits at Stage II versus fruits at early Stage II, 308 proteins (69%) had the same expression pattern therefore referred as "matching" (Figure 5a). In the comparison between fruits at Stage III versus Stage II 51% of the shared proteins displayed similar expression pattern and the rest fell under the "weak matching" category (Figure 5a). "Weak matching" refers to proteins showing significant expression changes with one method while showed no significant expression differences when analyzed with the other method (Figure 5b-d). Only few proteins, 1 and 16, showed contradicting expression patterns in the comparisons between Stage II versus early Stage II and between Stage III and Stage II, respectively.  The high percentage of proteins shared by dMS and SC that show the same expression pattern serves also as a strong validation for protein expression.

### Changes in protein expression during fruit development

Label-free LC-MS/MS analysis of juice sac cells indicated significant changes in protein synthesis during fruit development (Table 2). Changes in the expression of 1834 and 1004 iCitrus accessions during fruit development were identified by dMS and SC, respectively. These numbers consisted of accessions identified by the four types of comparisons conducted (Stage II vs. early Stage II, Stage III vs. Stage II, membrane-bound proteins and soluble), and proteins appearing at more than one stage of development were only counted once. In most cases, the discrepancies between the two methods were due to differences on the bioinformatics associated with dMS and SC workflows (see Discussion).

**Table 2:** Functional classification of proteins identified by dMS and SC workflows (see Experimental Procedures) after search of the iCitrus database.

	Stage II vs. early Stage II								Stage III vs. Stage II							
	Up		No Change		Down		Total		Up		No Change		Down		Total	
	
Functional category	dMS	SC	dMS	SC	dMS	SC	dMS	SC	dMS	SC	dMS	SC	dMS	SC	dMS	SC
Cell Cycle	0	0	1	1	3	1	4	2	0	0	0	2	0	0	0	2
Cell Wall	18	2	8	3	8	4	34	9	37	4	3	3	3	10	43	17
Energy	26	4	23	11	35	24	84	39	24	10	12	20	4	2	40	32
Heat Shock/Chaperone	66	25	40	15	29	8	135	48	80	21	55	24	0	10	135	55
Metabolism	86	34	87	44	115	38	288	116	238	53	91	111	5	27	334	191
Oxidative processes	32	11	39	11	34	11	15	33	90	20	18	24	6	5	114	49
Processing	9	15	34	16	63	14	106	45	62	26	40	35	7	16	109	77
Signaling	30	16	23	16	36	16	89	48	39	7	14	22	11	11	64	40
Structure	48	2	13	5	20	10	81	17	25	1	10	2	30	8	65	11
Trafficking	14	6	32	16	41	14	87	36	40	11	39	24	0	1	79	36
Transcription	4	2	26	12	24	12	54	26	33	4	2	13	3	6	38	23
Translation	65	10	35	28	113	32	213	70	43	5	87	39	10	28	140	72
Transport	29	6	15	16	43	24	87	46	70	12	29	24	0	1	99	37
Unknown	24	23	50	28	53	30	127	81	69	28	28	49	7	24	104	101
Sum.	451	156	426	222	617	238	1494	616	850	202	428	392	86	149	1364	743

Proteins were classified into 14 major groups and are represented according to fruit development stages comparisons according to the method used (dMS and SC).

A significant number of proteins (772 and 560) were identified and classified as "not changed" by dMS and SC, respectively (Table 2). Although these proteins were found to match differentially expressed peptides, did not pass the statistical threshold. Although not differentially expressed, the identification of these proteins provides valuable information because: (i) they are proteins that are active during fruit development; (ii) they strengthen the confidence in the identification of the same peptides in other comparisons [39]. Here, we have classified the fruit proteins into 14 major functional groups (Table 2). In general, the expression of a large number of proteins identified decreased during the transition from early Stage II to Stage II of development (617 were down-regulated and 451 were up-regulated). This trend reversed during the transition from Stage II to Stage III where 850 proteins were up-regulated and 86 were down-regulated (Table 2). Most of the up-regulated proteins belonged to Metabolism, Processing, Oxidative processes, Trafficking, Transcription and Transport.

### Changes in protein associated with vesicular trafficking during fruit development

In order to illustrate similarities and disparities between dMS and SC for the quantitation of protein changes during fruit development, we analyzed changes in proteins associated with vesicular trafficking and protein movements. The global changes in protein profiles and the metabolic processes associated with the quantitative protein changes during fruit development will be presented and discussed elsewhere (Katz *et al*., in preparation).

In this study, many small G-proteins and other proteins associated with a large number of cellular processes such as vesicle formation; vesicular traffic and docking, etc. [45-47] were found to be differentially expressed during fruit development (Tables 2, 3).

**Table 3:** Vesicular trafficking-related proteins identified by dMS and SC.

				Stage II (55 mm) vs. early stage II (35 mm)					Stage III (80 mm) vs. Stage II (55 mm)				
				dMS		SC			dMS		SC		
Gene Family	Annotation	iCitrus ID	Blast Hit TAIR ID	Peptides	Ratio	Bayes Factor	Fold Change	Direction*	Peptides	Ratio	Bayes Factor	Fold Change	Direction*
Rab	RABA1a/ARA2	5282	AT1G06400	3	0.03	--	--	--	2	2.85	--	--	--
	RABA1d/Rab11B	50939	AT4G18800	7	0.025	1.34	1.52	0	--	--	0.95	1.53	0
	RABA1f	23943	AT5G60860	5	0.11	465	31.9	-1	--	--	0.8	1.2	0
	RABA2a/Rab11C	33548	AT1G09630	6	0.72	63362	23	-1	2	7.43	31.8	4.5	1
	RABA2b	27900	AT1G07410	3	0.026	3302	28	-1	--	--	--	--	--
	RABB1b/Rab2C	28361	AT4G35860	--	--	--	--	--	3	33.41	--	--	--
	RABB1c	57271	AT4G17170	5	0.11	72	3.2	-1	4	28.6	1555	5.02	1
	RABD1/FP8	44137	AT3G11730	3	0.33	395	17.8	-1	--		0.9	1.45	0
	RABD2a/Rab1b	22194	AT1G02130	3	0.07	1472	24	-1	2	31.24	62.5	5.00	1
	RABD2b/Rab1A	21238	AT5G47200	2	0.04	--	--	--	--	--	--	--	--
	RABE1a/Rab8	55887	AT3G53610	3	0.03	54.9	3.6	-1	--	--	1.04	1.5	0
	RABE1e/Rab8E	58806	AT3G09900	2	0.04	--	--	--	--	--	--	--	--
	RABE1c/Rab8/ARA-3	21701	AT3G46060	4	1.06	8.8	1.8	0	2	3.48	0.82	1.2	0
	RABG3d	44916	AT1G52280	2	0.01	--	--	--	--	--	--	--	--
	RABG3f/Rab7B	30351	AT3G18820	3	0.006	0.8	1.3	0	2	6.94	2.16	1.79	0
	RABH1b/Rab6A	53105	AT2G44610	5	0.07	478	18.3	-1	--	--	0.75	1.2	0
	RABH1e	30604	AT5G10260	2	0.05	--	--	--	--	--	--	--	--
Arf	ARLA1c	26509	AT3G49870	3	0.79	0.58	1.13	0	2	4.49	1.07	1.67	0
	ARFA1e	422	AT3G62290	3	0.13	--	--	--	3	26.62	--	--	--
	ARFA1f	22081	AT1G10630	5	0.15	--	--	--	6	19.71	--	--	--
	SAR1c	34375	AT4G02080	--	--	--	--	--	3	4.91	--	--	--
Ran	STL2P/SEC12P-Like	54385	AT2G01470	--	--	2319	5.37	1	--	--	0.67	1.04	0
	RANBP1	42600	AT5G58590	5	0.5	3.4	2.8	0	7	129.80	0.6	1.98	0
	RANBP1b	2905	AT2G30060	--	--	1.3	4.2	0	5	130.08	280	22.3	1
	RAN3	57970	AT5G55190	2	0.87	0.64	1.82	0	7	20.25	3.7	1.67	0
Rho	GP3/ROP4	29311	AT1G75840	2	0.28	--	--	0	--	--	--	--	--
GDI	GDI1	34016	AT2G44100	--	--	1.58	2.18	0	3	5.00	0.73	1.13	0
	GDI2-like	876	AT5G09550	--	--	--	--	--	2	3.11	--	--	--
VAMP/R-SNAREs	SEC22	58654	AT1G11890	2	0.20	26.9	13.6	-1	--	--	1.06	1.46	0
	VAP27-1	54676	AT3G60600	2	0.77	--	--	--	--	--	--	--	--
	VAMP713	23669	AT5G11150	2	1.41	--	--	--	--	--	--	--	--
Qa-SNAREs	SYP132 (syntaxin 132)	12539	AT5G08080	--	--	3	6.2	0	2	3.21	1.25	1.65	0
	VAM3 (syntaxin 22)	37248	AT5G46860	--	--	1.35	1.86	0	--	--	4.5	2.3	0
Qb-SNAREs	VTI11	24253	AT5G39510	2	0.05	0.65	1.37	0	--	--	0.5	1.2	0
Qc-SNAREs	SYP71 (SYNTAXIN)	27696	AT3G09740	--	--	2.17	4.34	0	--	--	--	--	--
	ALPHA-SNAP2	2375	AT3G56190	--	--	1.00	1.00	0	--	--	6.41	7.6	0
ESCRT III	SNF7.1	35782	AT4G29160	4	1.31	4.88	2.99	0	7	597.36	106	5.5	1
Dynamin	ADL6 (dynamin-like protein 6)	36589	AT1G10290	--	--	--	--	--	2	6.63	--	--	--
others	SEC61	35474	AT1G29310	3	0.10	171.70	17.65	-1	--	--	1.00	1.4	0
	SEC14	30056	AT1G72160	5	3.42	37.17	13.65	1	16	12.92	4.8	1.5	0
	PATL2	35474	AT1G22530	3	0.104	170.5	17.65	-1	3	6.83	1.04	1.43	0
	SCAMP	34675	AT1G32050	3	1.70	1.50	1.55	0	2	4.11	0.74	1.18	0
	SC3 (secretory carrier 3)	30772	AT1G61250	2	0.98	2.9	6.12	0	--	--	1.00	1.02	0
	SYT1	45523	AT2G20990	4	8.7	0.3	1.5	0	4	2.64	1.2	1.6	0
	COP-I coatomer, B-COPα	29871	AT2G21390	2	0.4	0.6	1.22	0	3	11.47	1.31	1.73	0
	RTNLB3 reticulon	42672	AT1G64090	2	0.53	2.1	3.7	0	2	2.31	0.72	1.48	0
	RTNLB5 reticulon	13580	AT2G46170	--	--	0.97	1.02	0	--	--	4.35	2.97	0
	RTNLB6 reticulon	22293	AT3G10260	--	--	1.24	1.4	0	2	36.56	187.81	5.76	1
	clathrin heavy chain	57153	AT3G08530	--	--	83.92	13.36	-1	--	--	0.76	1.17	0
	clathrin heavy chain	60690	AT3G08530	2	0.05	63.7	13.65	-1	--	--	46.45	10.98	-1
	Clathrin light chain	51128	AT2G20760	2	119	2300591	11.55	1	2	1.8	0.8	1.2	0
	Clathrin light chain	21278	AT3G51890	2	118	1511	6.2	1	--	--	1.63	1.57	0
	myosin heavy chain	38388	AT4G31340	--	--	0.6	1.00	0	--	--	0.75	1.3	0
	myosin heavy chain	37311	AT1G06530	3	0.02	156	16	-1	--	--	1.1	1.4	0

* The column "direction" under spectral counting measurement represent expression direction, 1 = up-regulated, 0 = no change, -1 = down-regulated.

Proteins identified by dMS were considered to be upregulated when expression fold > 2, not changed when 0.5 < fold < 2 and down-regulated when fold change was < 2. For SC Bayes factor of > 10 was used for significance difference.

Several small G-proteins belonging to the sub-families RAB, ARF, RHO and RAN were differentially expressed during fruit development. For example, proteins belonging to the RAB-like sub-family (nomenclature according to Vernoud *et al*., [48]); RABA1a, RABA1 d, RABA1f, RABA2a, RABA2b, RABB1b, RABB1c, RABD1, RABD2a, RABD2b, RABE1a, RABE1c, RABE1e, RABG3 d, RABG3f, RABH1b and RABH1e were found to be differentially expressed (Table 3).  During the transition between early Stage II to Stage II most of this group of proteins was down-regulated according to dMS, except for RABA2a and RABE1c. Similarly to dMS, SC showed that RABA1f, RABA2a, RABA2b, RABB1c, RABD1, RABD2a, RABE1a and RABH1b, were down-regulated while no changes were detected in RABA1 d, RABE1c and RABG3f. During the transition from Stage II to Stage III, RABA1a, RABA2a, RABB1b, RABB1c, RABD2a, RABE1c and RABG3f were shown to be up-regulated by dMS (Table 3). SC detected up-regulation only for RABA2a, RABB1c and RABD2a at these stages. Few members of the ADP-ribosylation factor (ARF) were also found to be differentially expressed during fruit development. ARFA1e and ARFA1f were down-regulated during the transition from early Stage II to Stage II. On the other hand, ARLA1c remained unchanged (Table 3). ARFA1e, ARFA1f, ARLA1c and SAR1 provide another example of the difference in accuracy between dMS and SC. While dMS indicated that these four proteins were up-regulated during the later stages of fruit development (Table 3), SC indicated no change.  Four members of the RAN family, SEC12p, RANBP1, RANBP1b and RAN3 were identified in this study. The expression of RAN3 remained unchanged during the early stages of fruit development (as shown by both dMS and SC) but was up-regulated during the later stages. Both dMS and SC indicated that the expression of RANBP1b was up-regulated during the later stages of fruit development while only dMS showed up-regulation of RANBP1. SEC12p was up-regulated during the early stages and remained unchanged during the later stages of fruit development.

Among the members of the RHO family, ROP4 was down-regulated at earlier stages of development (Table 4). Interestingly, dMS showed that two RAB GDI (GDP-RAB dissociation inhibitors), GDI1 and GDI2-like were up-regulated during the later stages of fruit development while only GDI1was identified by SC. Three R-SNAREs were identified; SEC22 that was down-regulated during the transition from early Stage II to Stage II, VAMP27-1 and VAMP713 were identified but were not found to be differentially expressed. Five Q-SNAREs were identified but only VTI11 (Qb-SNARE) was found to be down-regulated during early stages of development while SYP132 (Qa-SNARE, syntaxin) was found to be up-regulated during late stages of development. SNF7, a component of the endosomal ESCRT III complex that functions in cargo recognition and sorting [49], was up-regulated during the late stages of development. Additional proteins related to vesicular trafficking such as dynamin, COP-I coatomer, reticulon 3 and 6, and proteins related to secretory membrane carriers such as SEC14, PATL2 and SYT1 were up-regulated during the late stages of fruit development, while SEC 14, SYT1,  and light chain of clathrin were up-regulated during the transition from early Stage II to Stage II. Heavy chain of clathrin was down-regulated throughout development (Table 3).

**Table 4:** Structure-related proteins identified by dMS and SC.

			Stage II (55 mm) vs. early stage II (35 mm)					Stage III (80 mm) vs. Stage II (55 mm)				
			dMS		SC			dMS		SC		
Annotation	iCitrus ID	Blast Hit TAIR ID	Peptides	Ratio	Bayes Factor	Fold Change	Direction*	Peptides	Ratio	Bayes Factor	Fold Change	Direction*
TUB1 (tubulin beta-1)	33157	AT1G75780	4	0.024	--	--	--	2	0.06	--	--	--
TUA3 (tubulin alpha-3)	46363	AT5G19770	4	0.07	--	--	--	--	--	--	--	--
TUA4 (tubulin alpha-4)	22742	AT1G04820	10	0.025	24.68	2.83	-1			1473619	13.49	-1
TUA4 (tubulin alpha-4)	28975	AT1G04820	10	0.025	--	--	--	4	0.16	--	--	--
TUA4 (tubulin alpha-4)	37760	AT1G04820	8	0.026	884.12	10.19	-1	3	0.7	0.6	1.4	0
TUB5 (tubulin beta-5)	30386	AT1G20010	9	0.02	17.65	4.6	-1	4	0.41	527	9.15	-1
TUB5 (tubulin beta-5)	32879	AT1G20010	9	0.04	3125	46.15	-1	--	--	--	--	--
TUB5 (tubulin beta-5)	50323	AT1G20010	10	0.02	27.89	14.3	-1	3	0.4	16293	33.34	-1
TUB5 (tubulin beta-5)	51971	AT1G20010	10	0.025	537	26.95	-1	4	0.39	12048	32.23	-1
TUB5 (tubulin beta-5)	53023	AT1G20010	9	0.033	3784	49.2	-1	--	--	--	--	--
TUA6 (tubulin alpha-6)	2232	AT4G14960	7	0.044	--	--	--	--	--	--	--	--
TUA6 (tubulin alpha-6)	48045	AT4G14960	4	0.025	--	--	--	2	21.7	--	--	--
TUB6 (tubulin beta-6)	54455	AT5G12250	6	0.027	--	--	--		2.18	--	--	--
TUB7 (tubulin beta-7)	54189	AT2G29550	2	0.6	--	--	--	2	2.19	--	--	--
TUB8 (tubulin beta-8)	55174	AT5G23860	5	0.022	--	--	--	3	45.85	--	--	--
ACT1 (actin 1)	45172	AT2G37620	3	0.3	--	--	--	3	22.49	--	--	--
ACT7 (actin 7)	3401	AT5G09810	6	0.025	42.61	2.24	-1	--	--	--	--	--
ACT7 (actin 7)	31765	AT5G09810	6	0.22	--	--	--	5	17.50	--	--	--
ACT8 (actin 8)	57479	AT1G49240	13	0.04	20.5	2.12	-1	8	21.93	20.36	2.12	1
ACT11 (actin 11)	51444	AT3G12110	4	0.26	--	--	--	3	27.16	--	--	--
CAM5 (Calmodulin 5)	18478	AT2G27030	--	--	7135	1.85	-1	--	--	32767	2.69	-1
CAM5 (Calmodulin 5)	51416	AT2G27030	7	0.15	--	--	--	--	--	--	--	--
KIS (KIESEL)	26013	AT2G30410	--	--	51.78	4.89	-1	--	--	48.8	8.36	-1
VLN3 (VILLIN 3)	11096	AT3G57410	--	--	1.19	3.00	0	--	--	1.55	1.74	0
microtubule associated protein (MAP65/ASE1)	38803	AT4G26760	4	0.03	95.54	28.3	-1	--	--	135.6	13.6	-1
PFN1/PRF1 (PROFILIN 1)	4145	AT2G19760	2	1.36	--	--	--	2	15.81	0.80	1.14	0
PFN3/PRF3 (PROFILIN 3)	23065	AT5G56600	3	1.47	0.85	1.4	0	2	15.81	1.4	3.8	0
PRF5 (PROFILIN5)	5851	AT2G19770	--	--	0.9	1.34	0	--	--	0.6	1.03	0
ADF4 actin depolymerizing	7407	AT5G59890	--	--	6.8	5.2	0	--	--	28.89	10.4	-1

* The column "direction" under spectral counting measurement represent expression direction, 1 = up-regulated, 0 = no change, -1 = down-regulated.

Proteins identified by dMS were considered to be up-regulated when expression fold > 2, not changed when 0.5 < fold < 2 and down-regulated when fold change was < 2. For spectral counting Bayes factor of > 10 was used for significance difference.

Differential protein expression was also found in other important groups of proteins, actins and tubulins, key factors in trafficking, cell division and enlargement [50]. TUB1, TUA3, TUA4, TUB5, TUA6, TUB6 and TUB8 were down-regulated in the transition from early Stage II to Stage II (Table 4). TUB1, TUA4, TUB5, TUA6 and TUB6 were down-regulated further during the transition from Stage II to Stage III while TUB7 and TUB8 were up-regulated during this transition. Actins, driving vesicular movement towards their destination, showed significant changes during fruit development (Table 4). ACT1, ACT7, ACT8 and ACT11 were down-regulated during the transition from early stage II to stage II and were up regulated during the transition from stage II to stage III (Table 4).

Down-regulation of other proteins related to the vesicle movements such as CaM5 (which binds to the motor protein kinesin [51,52] and myosin were detected (Table 4). Profilins, PFN1, PFN3 and PFN5, involved in actin polymerization and cytoskeleton organization did not change during the transition from early Stage II to Stage II, but PFN1 and PFN3 were up-regulated during the transition from Stage II to Stage III. Another protein, ADF4, involved in actin de-polymerization was down regulated during the transition to Stage III. Microtubule Associated Protein 65 (MAP65) and KIS (Tubulin cofactor A) involved in tubulin complex assembly and cell division [53,54], were down-regulated throughout fruit development (Table 4).

Transporters play a crucial role in cell growth and homeostasis, especially in specialized solute accumulating cells such as citrus juice cells. As expected, many changes in transporters protein expression were noted during fruit development (Table 5). During the transition from early Stage II to Stage III, there was a significant down-regulation of subunits of lysosomal ATPases and cation transporters associated with K^+^- and Na^+^-coupled transport. On the other hand, only one plasma membrane-bound ATPase displayed down-regulation (similar to AHA8), while those similar to AHA2, AHA4 and AHA10 were not significantly changed. In general, these changes were noted using both dMS and SC. Most of the proteins that were down-regulated during the transition from early to Stage II, were up-regulated during the transition from Stage II to Stage III (Table 5), suggesting their role during fruit expansion. Similar results were seen with mitochondrial-bound proteins such as ACP4, ADP/ATP carriers and others. Two tonoplast monosaccharide transporters, TMT1and TMT2 were up-regulated during the transition from early to stage II and TMT2 was further up-regulated during the later stages of fruit development. A dicarboxylate/tricarboxylate carrier was up-regulated throughout development. Plasma membrane water channels PIP1B/PIP1;2, TMP-C/PIP1;4, PIP2;8/PIP3B and PIP2;5/PIP2 D were down-regulated during the transition from early to Stage II according to SC (Table 5).

**Table 5:** Transport-related proteins identified by dMS and SC.

			Stage II (55 mm) vs. early stage II (35 mm)					Stage III (80 mm) vs. Stage II (55 mm)				
			dMS		SC			dMS		SC		
Annotation	iCitrus ID	Blast Hit TAIR ID	Peptides	Ratio	Bayes Factor	Fold Change	Direction*	Peptides	Ratio	Bayes Factor	Fold Change	Direction*
Ca^2+ ^-ATPase	45644	AT1G07670	--	--	1.2	2.9	0	2	20.88	27.99	4.99	1
Ca^2+^/Na^+ ^exchanger	47787	AT1G53210	2	0.27	14.27	3.00	-1	2	9.04	0.71	1.15	0
KEA2 (K^+^/H^+ ^antiporter)	36862	AT4G00630	2	0.40	1.41	1.75	0	--	--	0.8	1.2	0
porin, putative; voltage-dependent anion channel	23759	AT3G01280	2	0.19	13.9	6.34	-1	2	6.09	15.91	4.81	1
DET3 C subunit C (V-type H^+^- ATPase)	58235	AT1G12840	3	0.27	45.53	3.26	-1	--	--	1.2	1.48	0
AVP1 (vacuolar H+-PPiase)	36100	AT1G15690	4	2.31	1.1	1.2	0			2.76	1.63	0
VHA-A	27256	AT1G78900	13	0.04	1011648	3.99	-1	12	9.94	38.82	2.2	1
VHA-A3 (V-ATPase)	49974	AT4G39080	4	0.44	--	--	--	3	4.01	--	--	--
V-type ATPase subunit B1	3683	AT1G76030	8	0.05	--	--	--	5	13.78	--	--	--
V-type ATPase subunit B2	62399	AT4G38510	11	0.06	--	--	--	9	11.25	--	--	--
V-type ATPase subunit B3	37671	AT1G20260	11	0.06	140.60	2.13	-1	9	11.25	1004	2.64	1
V-type ATPase subunit D	23422	AT3G58730	--	--	34.4	5.4	-1	--	--	1.55	1.45	0
V-type ATPase subunit E1	52391	AT4G11150	--	--	19.83	3.22	-1	2	5.12	7.23	1.94	0
VMA10 (V-type ATPase 10); Subunit G	28722	AT3G01390	3	0.28	36.6	2.8	-1	--	--	0.15	1.78	0
V-type ATPase subunit H	38700	AT3G42050	6	0.25	36.6	2.82	-1	4	13.57	0.58	1.06	0
AHA2 (H^+^-ATPase 2)	50370	AT4G30190	3	0.53	0.98	1.23	0	4	11.79	1.75	1.49	0
AHA4 (H^+^- ATPase 4)	28397	AT3G47950	3	1.14	11055	20.4	-1	3	7.40	0.9	1.5	0
AHA8 (H^+^- ATPase 8)	56228	AT3G42640	2	0.35	--	--	--	2	13.60	--	--	--
AHA10 (Autoinhibited H^+^- ATPase isoform 10)	56764	AT1G17260	4	1.13	5.8	1.55	0	4	41.48	1.36	1.19	0
H^+^- ATPase	42025	AT3G28710	2	0.11	335	18.72	-1	2	17.27	22.04	4.37	1
TMT1 (tonoplast monosaccharide transporter1)	44853	AT1G20840	3	24.79	3.0	1.1	0	--	--	382	5.1	1
TMT2, (tonoplast monosaccharide transporter 2	41259	AT4G35300	8	32.69	16121	2.64	1	6	15.26	9.5	1.9	0
GPT2 (glucose-6-phosphate/phosphate translocator 2)	44184	AT1G61800	2	0.10	28.2	12.3	-1	--	--	0.8	1.2	0
mannitol transporter	32978	AT2G16130	--	--	439	18.8	-1	--	--	5.8	3.2	0
LPT(lipid transfer protein)	45077	AT1G27950	2	0.01	5258.00	22.44	-1	--	--	--	--	--
LTP3	58907	AT5G59320	--	--	2.86	4	0	--	--	7447	21.8	-1
PDR6/PDR6 (pleiotropic drug resistance 6); ATPase	26072	AT2G36380	--	--	4.5	7.11	0	--	--	49.8	3.9	1
PDR11/PDR11 (pleiotropic drug resistance 11); ATPase	52044	AT1G66950	--	--	--	--	--	2	11.74	1.18	1.56	0
PDR12/(PLEIOTROPIC DRUG 12); ATPase	19314	AT1G15520	--	--	0.98	1.03	0	2	14.97	5.44	3.46	0
MAPR2 (membrane-associated progesterone binding protein 2)	28962	AT2G24940	3	1.62	1.57	1.65	0	3	45.68	1.38	1.69	0
MRP4 (multidrug resistance-associated protein 4)	46730	AT2G47800	2	1.36	2.62	2.64	0	5	58.12	19.3	2.6	1
PGP7 (P-GLYCOPROTEIN 7); ATPase	10552	AT5G46540	--	--	2.06	5.56	0	--	--	10.4	3.6	1
PIP1B (plasma membrane intrinsic protein 1;2); water transport	18285	AT2G45960	--	--	149.18	27.06	-1	--	--	--	--	--
PIP2;8/PIP3B (plasma membrane intrinsic protein 2;8); water channel	25444	AT2G16850	--	--	462	23	-1	--	--	0.9	1	0
PIP2;5/PIP2 D (plasma membrane intrinsic protein 2;5); water channel	41682	AT3G54820	--	--	635.26	39.02	-1	--	--	--	--	--
TMP-C (plasma membrane intrinsic protein 1;4); water channel	28678	AT4G00430	3	0.11	35.06	10.97	-1	--	--	2.76	2.70	0
protein transporter	51128	AT2G20760	3	30	0.2	1.6	0	2	1.80	0.84	1.22	0
protein transporter	21278	AT3G51890	2	118.94	1510	6.2	1	--	--	1.63	1.57	0
TOM20-3 (translocase of outer membrane)	33905	AT3G27080	4	0.45	0.6	1.16	0	3	4.75	18.02	2.97	1
ACP4 (acyl carrier 4)	25090	AT4G25050	3	0.34	3.1	2.27	0	--	--	13.4	7.8	1
AAC2 (ADP/ATP carrier)	53189	AT5G13490	8	0.27	16767	10.36	-1	5	21.36	1.01	1.02	0
AAC3 (ADP/ATP carrier)	26451	AT4G28390	10	0.22	1040	3.48	-1	6	76.16	32.95	2.75	1
mitochondrial phosphate transporter	44051	AT5G14040	4	0.10	10.8	3	-1	2	4.29	0.79	1.2	0
PDE120 protein import (Tic40)	57128	AT5G16620	2	0.34	--	--	--	--	--	--	--	--
di/tricarboxylate carrier	39221	AT5G19760	5	56.47	0.52	1.49	0	2	25.92	0.69	1.13	0
TOM22-V (translocase outer mitochondrial membrane)	38717	AT5G43970	--	--	160	17.8	-1	--	--	2.98	2.65	0
Lipocalin	23197	AT5G58070	2	29.74	44.2	4.3	1	3	10.49	40.34	2.3	1
												
												

* The column "direction" under spectral counting measurement represent expression direction, 1 = up-regulated, 0 = no change, -1 = down-regulated.

Proteins identified by dMS were considered to be up-regulated when expression fold > 2, not changed when 0.5 < fold < 2 and down-regulated when fold change was < 2. For spectral counting Bayes factor of > 10 was used for significance difference.

## Discussion

In this study we describe a label-free shotgun approach to establish a proteomics workflow for the identification of the protein changes occurring during citrus fruit development. We analyzed and compared juice sac cells extracted from fruits at three stages of development. The end of Stage I (early Stage II), characterized by extensive cell division; Stage II, where cell division ceases and the juice cell sacs expand with the accumulation of large amounts of solutes and water; and Stage III, where the fruit matures and ripens  [55,56]. It should be noted that it was practically impossible to extract juice sac cell proteins at Stage I (fruit diameter ≈10-15 mm) because at this stage the juice sac cells are not well developed.

Comparative proteomics studies in plants are still lagging behind studies done in mammalian cells and are predominantly performed by employing 2DE-gels [57]. Although differential proteomics studies employing label-free quantification have been published during the last few years [9,10,24], in plants these studies are scarce [26,43].

In order to employ an efficient proteomics study in citrus, a plant species lacking a full sequenced genome, we established a workflow that dealt with few of the problems arising from using a ESTs database. We created iCitrus, a database and interface that collected sequences from three different sources, HarvEST:Citrus http://harvest.ucr.edu/, NCBI's Citrus unigenes and NCBI's Citrus proteins http://www.ncbi.nlm.nih.gov/Taxonomy/Browser/wwwtax.cgi?mode=Info&id=2711&lvl=3&lin=f&keep=1&srchmode=1&unlock to create one unified database with reduced redundancy for mass spectra search. iCitrus was created to provide a compact database for the identification of citrus proteins and a more accurate quantitative expression measurements. The iCitrus interface enabled a fast identification of lists of accessions including Arabidopsis homologs, and the use of bioinformatics tools such as MapMan, AraCyc and Cytoscape (Katz *et al*. in preparation).

The iCitrus resource is essentially an interface that can be used to access pre-calculated Blast results. iCitrus itself does not make or summarize GO assignments based on rules that weight GO terms from various hits; this is the (perfectly reasonable) philosophy behind Blast2GO and related tools. We chose to allow users, instead of iCitrus, to determine if they trust and adopt particular annotations or not. We took this approach to allow individual users to use specific knowledge of protein families or taxonomical differences (i.e. Citrus versus Arabidopsis) to influence their interpretation of the BLAST results. In addition, there may be cases in which GO annotation is absent in the BLAST results against Arabidopsis or Viridiplantae, but a consensus could emerge from the descriptive text accompanying a hit. We think this combined approach of manual annotation with the assistance of pre-computed BLAST results is more effective when predicting functional information for a not well-annotated organism like Citrus.

Two widely used, but fundamentally different, label-free methods for quantification were used in this study; peak integration (dMS) and spectral counting (SC). For dMS, we used a two-fold change as a threshold for differential expression of the identified proteins [25] and a Bayes factor of 10 for spectral counting [58]. Such a stringent threshold is needed because the protein ratios are calculated by averaging the intensity weight of peptide ratios, and because the number of peptides identifying each protein is highly variable. In most cases, both methods identified similar proteins with some discrepancies (Figure 4a). These discrepancies derived from the way SIEVE (for dMS) and Scaffold (for SC) handled the peptides information. Scaffold is able to identify peptides in similar proteins and group them together, thus identifying database redundancy, on the other hand, SIEVE does not group similar proteins. When we compare the number of identified proteins by the two methods using the corresponding Arabidopsis homologs of each iCitrus accession identified (Figure 4b) the differences decreased significantly, particularly for dMS (Figure 4). Yet, additional redundancy could arise from possible gene families in Citrus. The wide range of Citrus species used to create HarvEST:Citrus database including Citrus *sinensis*, Citrus *paradise*, Citrus ***unshiu***, C. *reticulata*, C. *jambhiri*, C. *aurantium*, C. *clementina*, C. *macrophylla *and Poncirus *trifoliate*, consists of sequences that are similar but not identical therefore were not screened out from the iCitrus dataset. In addition, some of the sequences in the database that might originate from the same unigene did not overlap therefore could not be assembled, contributing to the difference in number of proteins identified (Table 1).

Currently, non-overlapping sequences cannot be assembled until more ESTs can be produced to cover the missing gaps or until the Citrus genome is fully sequenced [59]. A significant number of proteins (144 in dMS and 118 in SC in the Stage II vs. early Stage II comparison, and 119 in dMS and 255 proteins in SC, in the Stage III vs. Stage II comparison) were identified by only one of the methods due to the inherent differences of dMS and SC workflows. SEQUEST and SIEVE (dMS workflow) use protein probability cut-off based on false discovery rate (FDR) according to the Decoy method [60]. X!Tandem, Scaffold and Qspec (SC workflow) use peptide identification probability criteria as specified by the Peptide Prophet algorithm [61]. The different workflows affect some of the proteins identification. The performance of the SC method depends strongly on the depth of the MS/MS sampling because ratios by SC are most significant for proteins with large numbers of product ion spectra, while ratios by dMS are most significant for proteins with large numbers of overlapping peptide ions [25]. This also explains the higher percentage of proteins that were found to be significantly different by dMS and not significant by SC (Figures 3, 5a). Therefore, dMS provides more accurate measurements of compared samples while SC is faster and easier to use. Our data show that dMS is more accurate in measuring differences in protein expression [25]. dMS provide rich information of the LC-MS data but requires a massive computational effort to be spent on processing the data including background filtering, peak frame detection and alignment [62,63]. Spectral counting is conceptually simpler and can be as sensitive as dMS in terms of detection range while retaining linearity [25,30,64]. Nevertheless, SC is less accurate in detecting differences in protein expression, in particular for less abundant proteins. Our results clearly show that the integrated use of both methods for quantification increases the power for detecting changes in shotgun proteomics experiments, and that both methods should be use in combination to gain insight of the complex protein network and a complete identification of its components.

Changes in a large number of small GTPases were identified during citrus fruit development. The expression of a relatively large number of members of the RAB, ARF, RHO and RAN families of small GTPases changed during the different stages. Although we cannot assign specific roles to all of these proteins, they clearly indicate a different role(s) of these members during the stages of citrus juice sac cell development. Vesicular trafficking is essential for fruit development [65-67]. During the Stage I there is intensive cell division [56]. Cytoskeleton elements (actins, tubulins, etc.) together with small G-proteins and coatomer complexes are vital to cell division, cell plate formation, cell polarity, etc. [68]. The expression of many of these proteins decreased during the transition from early Stage II to Stage II. This correlated well with the attenuation of cell division in the growing fruit and the prevalence of cell expansion. This notion was reinforced by the notable increase in expression of other small GTPases, auxiliary proteins and cytoskeletal components. Similar to the small G-proteins, changes in the expression of proteins associated with vesicular movements, docking and fusion were seen. In addition to different SNAREs (Qa, Qb, Qc, syntaxins, etc.), there was changes in COPI coatomers, clathrin, dynamin, and others suggesting the occurrence of endocytosis, exocytosis and vesicular trafficking during fruit development. Notably, while the expression of plasma membrane-associated H^+^-ATPases did not change during the early stages of development, changes in endosomal-associated H^+^-ATPases (V-type) paralleled the changes seen in the secretory and vesicular trafficking machinery.  V-type ATPases and organellar acidification is essential for vesicular trafficking along exocytotic and endocytotic pathways [69,70].

Although significant changes in sugar contents and sugar homeostasis are expected during fruit development [71,72], changes in expression of only two putative vacuolar monosaccharide transporters (TMT1 and TMT2) were noted. A plausible explanation is that the expression of other sugar transporters did not change (although they could have been modified by post-translational mechanisms). In support of this notion, Etxeberria *et al*. [73,74] demonstrated a mechanism of sugar transport into the juice sac cells and sucrose into the vacuoles that is mediated by endocytosis and intracellular vesicular trafficking. The protein inventory developed in this work, provides a preliminary glance at the function(s) of these proteins during the different stages of fruit development and in particular during cell division (Stage I, early Stage II) and cell expansion (Stage II) and assimilate mobilization, sugar accumulation and processes regulating fruit maturation and ripening.

In conclusion, we developed a workflow for the analysis and identification of proteins during fruit development in citrus, a non-model plant, using comparative label-free shotgun proteomics. We established iCitrus, a comprehensive sequence database by merging three major sources of sequences and improving the annotation of existing unigenes. iCitrus provided a useful bioinformatics tool for the high throughput identification of citrus proteins. Two methods for label-free based shotgun proteomics were used and compared; peak integration (or differential mass-spec) and spectral counting. We have identified approximately 1500 citrus protein accessions expressed in fruits and quantified their expression changes during fruit development. Our results showed that both methods can provide significant information on protein changes, with dMS providing higher accuracy. Our results clearly suggest that dMS and SC are matching, broadening the identification spectrum and providing complementary data on the change trends during the particular processes being compared.

## Methods

### Plant material, protein extraction and precipitation

Orange Navel fruits at three different developmental stages, early stage II (35 mm in fruit diameter), stage II (55 mm) and stage III (80 mm) [55] were obtained from the Lindcove Research Center, University of California, Exeter, CA.  Juice sacs were collected from at least 20 fruits and pooled at each stage. Two independent biological repetitions from two consecutive years were used and  proteins were isolated as described before [18]. Soluble proteins were precipitated using a chloroform/methanol extraction method as described by Wessel and Flugge [75]. The samples were resuspended with 100 μl of 1% Acetonitrile and sonicated for 10 min and centrifuged at 10,000 *g *for 3 min.  The supernatant  was spin-dialyzed into 50 mM ammonium-bicarbonate (AMBIC), then prepared for MS analysis using standard reduction, alkylation, and tryptic digest procedures [76].  Dichloromethane was added (50/50 v/v with aqueous digest) before vortexing for 1 min.  Samples were centrifuged for 5 min at 10,000 *g *in a microcentrifuge and the upper layer-containing peptides dried down and the peptides resolubilized in 2% acetonitrile/0.1% trifluoroacetic acid for LC-MS/MS analysis.

Membrane-bound proteins were spin-dialyzed into 50 mM AMBIC. An endo-polygalacturonanase (Megazyme) was employed to degrade pectins overnight at room temperature and the suspensions centrifuged and the pellets retained. Membranes were resolubilized in 50 mM AMBIC and digested with trypsin. The suspension was centrifuged 10 min at 10,000 *g *and the supernatant containing tryptic peptides retained.  Delipidation was performed with dichloromethane and the peptides resolubilized in 2% acetonitrile/0.1% trifluoroacetic acid for LC-MS/MS analysis.

### Mass Spectrometry and Data Analysis

Digested peptides were separated by reversed-phase chromatography using a Waters nanoACQUITY-UPLC system (Milford, MA), with a Waters BEH C_18 _1.7 μm, 100 μm × 10 cm column.  A binary solvent gradient was employed; buffer A was composed of 0.1% formic acid and buffer B composed of 100% acetonitrile (CAN).   The 120 min gradient consisted of the steps 2-45% buffer B in 40 min, 45-80% buffer B in 65 min, hold for 1 min, 80-2% buffer B in 4 min, then hold for 10 min. Separated peptides were analyzed in a Thermo-Scientific LTQ-FT Ultram mass-spectrometer (San Jose, CA) with a Michrom captive spray nano-electrospray ionization source at a flow rate of 2 μl/min. MS and MS/MS spectra were acquired using a top 4 method, where the 4 most abundant ions in the MS scan were selected for automated low energy Collision-induced Dissociation (CID) with a 30 s exclusion time and repeat count of 2. The FTMS scan was obtained for the m/z range 300-1400 Da at 50,000 resolution.  An isolation width of 2.5 Da was used for ITMS, and a normalized collision energy of 35% was used for the fragmentation.  Five technical repeats of each pooled sample (older *vs *younger fruit) were each analyzed by SIEVE using  blanks (washes) between each sample run.

### Protein Identification and Validation, dMS workflow

Tandem mass spectra were extracted with Xcalibur version 2.0.7. All MS/MS samples were analyzed using SEQUEST (Protein Discoverer 1.1; Thermo-Scientific, San Jose, CA).  SEQUEST was set up to search a FASTA file of the iCitrus Protein Database (see below), assuming the digestion enzyme trypsin. SEQUEST was searched with a peptide ion mass tolerance of 25 ppm and a fragment ion mass tolerance of 1.0 Da. Oxidation of methionine and iodoacetamide derivative of cysteine was specified in SEQUEST as possible modifications.  DTASelect software was used to filter out low score matching.  The filtering criteria consisted of  Cross-correlation (xcorr) values larger than 1.5 for single-charged ions, 2.2 for double-charged ions, and 3.3 for triple-charged ions, for both half or fully tryptic peptides. This resulted in a false discovery rate of less than  5% using a decoy search strategy.

### Differential Expression mass spectrometry, dMS workflow

Samples were analyzed using a Thermo Scientific LTQ-FT mass-spectrometer and a Michrom-Paradigm HPLC. Peptides were separated using a 200 μm × 15 cm Michrom Magic C18 reverse-phase column over 45 min using an acetonitrile gradient of 2%-60%. The mass-spectrometer was set to acquire spectra in standard top 3 method where 1 high resolution scan (100 K resolution) was acquired every sec with subsequent MS/MS spectra acquired in the LTQ simultaneously.

Samples were analyzed using SIEVE (Thermo Scientific, San Jose Ca). SIEVE is a label-free-differential expression package that aligns the MS spectra over time from different experimental conditions and then determines features in the data (m/z and retention time pairs) that differ across the different conditions. These differences were assigned using various statistics methods such as a P-Value and standard deviation and then sorted based on significance [10], based on the values obtained from the data of each biological replicate. Label free proteomic profiling was accomplished using SIEVE 1.3 (Thermo Scientific, San Jose, CA). The following parameters were set to align the retion time and generate the frames needed for abundance calculations. Alignment Parameters; Alignment Bypass = False, Correlation Bin Width = 1, RT Limits For Alignment = True, Tile Increment = 150, Tile Maximum = 300, Tile Size = 300, Time Threshold = 0.6. Frame Parameters; AVGCharge Processor = False, MS2 Corr Processor = False, M/Z Min = 300, M/Z Max = 1,400, Frame time Width (min) = 5.0 minutes, Frame M/Z width = 0.02 da, Search Window % = 50%, Retention Time Start = 5.0 min, Retention Time Stop = 110 min, Peak Intensity threshold = 50,000, Processor Modules = Isotagger V1.1, PCA V1.0. Significance was calculated within SIEVE using a standard T-test and results were filtered for a minimum of two peptides identified per protein (using the identification criteria stated in this method section) with frames having a p value of less than 0.05.

Tandem mass-spectra from peptide features that are considered differentially expressed across conditions are then searched using SEQUEST against iCitrus (see below). Search results were filtered for a False Discovery rate of 5% employing a decoy search strategy utilizing a reverse database [60].

### Protein Identification and Validation for Spectral counting

Tandem mass-spectra were extracted by Bioworks-3.3. Charge state de-convolution and de-isotoping were not performed. All MS/MS samples were analyzed using X! Tandem http://www.thegpm.org; version TORNADO (2008.02.01.2)). X! Tandem was set up to search the 62,415 entries of iCitrus (see below) assuming the digestion enzyme trypsin. X! Tandem was searched with a fragment ion mass tolerance of 0.40 Da and a parent ion tolerance of 25 ppm. Iodoacetamide derivative of cysteine was specified in X! Tandem as a fixed modification. Deamidation of asparagine, oxidation of methionine, sulphone of methionine, tryptophan oxidation to formylkynurenin of tryptophan and acetylation of the N-terminus were specified in X! Tandem as variable modifications. Different tandem MS programs were used (SEQUEST for dMS and X!Tandem for Spectral Counting) because of licensing restriction and limited access to SEQUEST that would have generated significant time delays in the data analysis. Nonetheless,  the use of SEQUEST or X!Tandem would have make little or no difference. In addition, in this report we aim at comparing overall methodology (i.e. dMS versus SC) and not their individual components.

### Criteria for protein identification for Spectral Counting

Scaffold 2.06.00 (Proteome Software Inc., Portland, OR) was used to validate MS/MS-based peptide and protein identifications. Peptide identifications were accepted if they could be established at greater than 80.0% probability as specified by the Peptide Prophet algorithm [61].  Protein identifications were accepted if they could be established at greater than 95.0% probability and contained at least 2 identified peptides.  Protein probabilities were assigned by the Protein Prophet algorithm [77]. Proteins that contained similar peptides and could not be differentiated based on MS/MS analysis alone were grouped to satisfy the principles of parsimony.

### Statistical Analysis for Spectral Counting

Unweighted Spectral counts for the identified proteins obtained from the samples corresponding to two consecutive growth seasons were exported from Scaffold and analyzed using QSpec [58] for significance analysis. Proteins were considered significantly different across sample conditions if QSpec reported a Bayes factor of > 10. This corresponds to a false discovery rate (FDR) of approximately 5%.

### Proteomics Data Set

The data associated with this manuscript may be downloaded from ProteomeCommons.org Tranche using the following hash:

Cf3G8KatEeCbDv2kV1Gnw4njaSYARJgmtyzYl+5764Gsbb/M3LX+/oo1zcHnHK1Gs0ukuBM5Rk+Q1t5hpia109pVPXkAAAAAAAAoLg==The hash may be used to prove exactly what files were published as part of this manuscript's data set, and the hash may also be used to check that the data has not changed since publication.

## Competing interests

The authors declare that they have no competing interests.

## Authors' contributions

EK, MGF, AS and EB conceived and designed the experiments. EK, MGF, RAE, and BSP performed the experiments. JNF and DL contributed to the design of iCitrus. EK, MGF, RAE, BSP, AS and EB analyzed the data. EK and EB drafted the manuscript. All authors read and approved the final manuscript.

## Supplementary Material

Additional file 1 **iCitrus database file in FASTA format**. Citrus sequences from UC Riverside HarvEST:citrus (C46 assembly), NCBI/citrus/unigenes and NCBI/citrus/proteins were used to creat iCitrus. Thedatasets were merged and identical sequences were filtered for redundancy (the longest sequences were kept). All sequences were blasted to TAIR, and separately to nr sequences belonging to taxa within *Viridiplantae*, in order to collect GO-term and descriptive annotations. The sequences are listed according to iCitrus accessions numbers followed with their HarvEST accession, NCBI\citrus\unigene accession or NCBI\Citrus\protein accession, Arabidopsis best homolog, annotation and amino acids sequence. Users can enter lists of citrus sequence ID's, which results in a table of ranked hits from a blast search of the citrus sequences against Arabidopsis or *Viridiplantae *sequences. ID's from 1 to 62415, representing the collected accessions, can be entered in the iCitrus interface (Figure 1). Each citrus ID received its own section of the result table and each ID (hits) to TAIR proteins is separated into two blocks, defined by the high scoring pair wise (HSP)-to-query coverage cutoff that can be set on the front page. All BLAST hits with e-values than 1E-4 are reported, and no hits below that cutoff occurred for a particular sequence, an empty list is returned. The TAIR ID (AGI number) and NCBI gi number of the Arabidopsis or *Viridiplantae *protein similar to the citrus sequence are shown next, including links to TAIR and NCBI.  Finally, GO annotations are listed when available. The final column "Annotation" contains TAIR-specific annotations that do not use the same terms as the Gene Ontology, but are available for the TAIR proteins. The data can be downloaded to any spreadsheet. Click here for file

Additional file  2 **Table S1. Conversion table into iCitrus**.  Conversion table  of HarvEST:Citrus, NCBI/Citrus/ESTs and NCBI/Citrus/Proteins accessions into iCitrus accessions. A complete list of all iCitrus accessions can be found in columns A (62,415 accessions). Column A consists of accessions from three databases: (1) NCBI/Citrus/Unigenes (accessions are numbered S#####) (2) HarvEST:Citrus ESTs (UC46_#####) (3) NCBI/Citrus/Proteins (#####) and column B consist of the corresponding iCitrus ID's. iCitrus ID's organized in ascending order. A list of accessions, originated from the three databases that were found to be clustered together is shown in columns D-F. Column D consists of accessions that were found to be clustered with other accessions and column E consists of accessions that clustered with accessions in column D. Column F consists of the corresponding iCitrus accessions of the clustered accessions appeared in columns D and E. A list of accessions that are found in the databases (NCBI and HarvEST:Citrus) but are shorter than 50 AA between stop codons, are shown in column H. These sequences were taken out of iCitrus database and cannot be found in the FASTA file (Additional File 1). Fast conversion table between the different sources of sequences can be found in columns L-O. Click here for file

Additional file  3 **Figure S1. Alignment and analysis of LC-MS/MS runs**. 10 replicate LC-MS/MS runs (5 per condition) aligned and analyzed using SIEVE. Several examples for high accuracy RT-XIC pairs are shown. (a) RT-XIC pair for early stage II in blue and stage II, in red. (b) A peptide significantly up-regulated in Blue, (c) a peptide that does not show a significant expression difference, and (d) a peptide significantly up-regulated in red. Click here for file
