# Supplementary figures and images for: A label-free differential quantitative mass spectrometry method for the characterization and identification of protein changes during citrus fruit development
Source: Proteome Sci. 2010 Dec 16;8:68. doi: 10.1186/1477-5956-8-68 (PMC3017515; doi:10.1186/1477-5956-8-68)

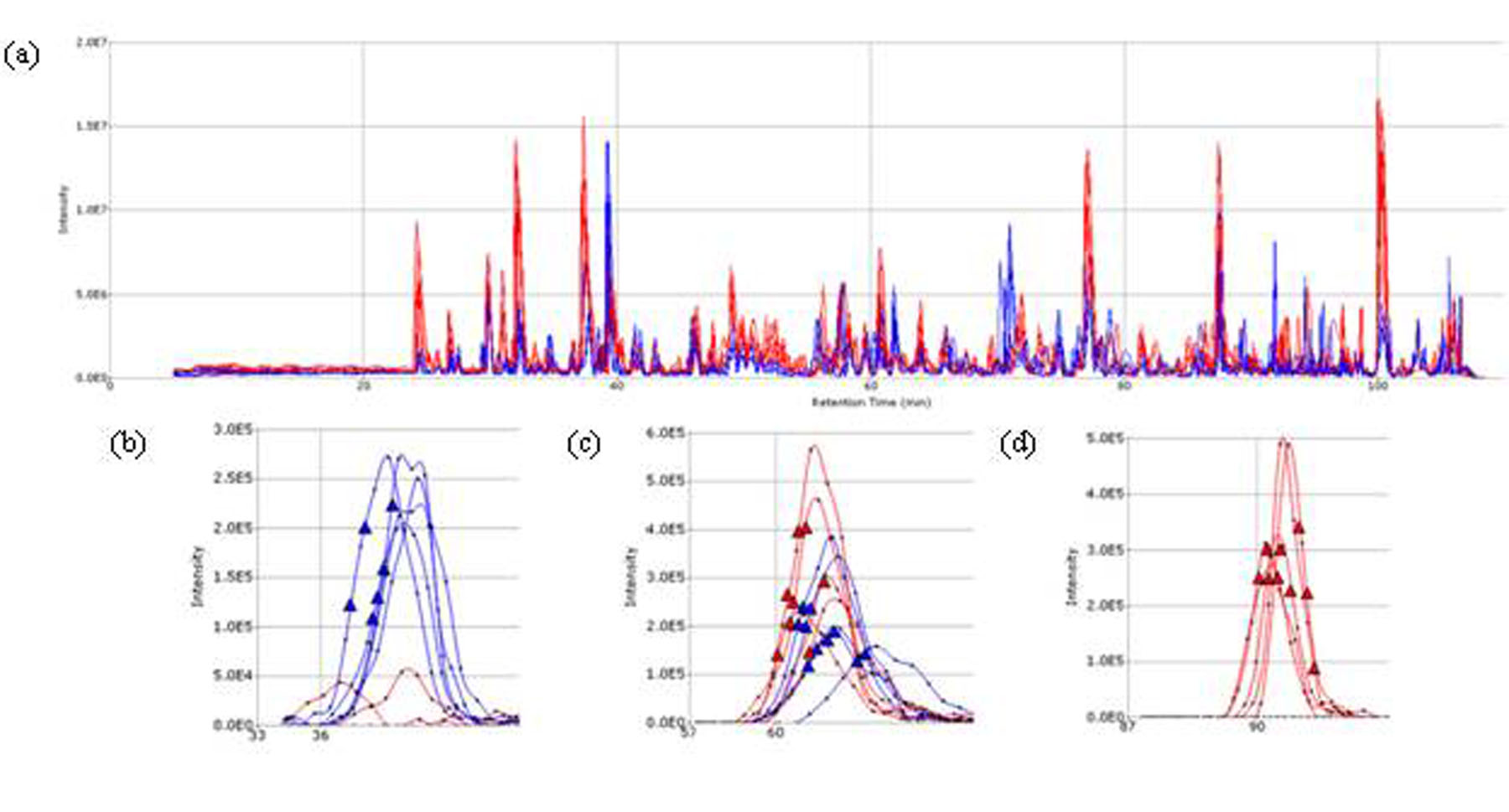

Supplement: Additional file 3 — Figure S1. Alignment and analysis of LC-MS/MS runs. 10 replicate LC-MS/MS runs (5 per condition) aligned and analyzed using SIEVE. Several examples for high accuracy RT-XIC pairs are shown. (a) RT-XIC pair for early stage II in blue and stage II, in red. (b) A peptide significantly up-regulated in Blue, (c) a peptide that does not show a significant expression difference, and (d) a peptide significantly up-regulated in red. [file 1477-5956-8-68-S3.JPEG]
